# Chloroplast Genome Comparison and Phylogenetic Analysis of the Commercial Variety *Actinidia chinensis* ‘Hongyang’

**DOI:** 10.3390/genes14122136

**Published:** 2023-11-27

**Authors:** Han Liu, Xia Liu, Chong Sun, Hong-Lei Li, Zhe-Xin Li, Yuan Guo, Xue-Qian Fu, Qin-Hong Liao, Wen-Lin Zhang, Yi-Qing Liu

**Affiliations:** 1 College of Landscape Architecture and Life Science, Chongqing University of Arts and Sciences, Chongqing 402160, China; liuhan0402@163.com (H.L.); zbgqsc1987@163.com (C.S.); lihonglei215@163.com (H.-L.L.); lizhexin_8903@163.com (Z.-X.L.); 15923370686@163.com (Y.G.); fxquick120@163.com (X.-Q.F.); lqhwisdom@163.com (Q.-H.L.); zhangwenlin@cqwu.edu.cn (W.-L.Z.); 2 College of Biology and Food Engineering, Chongqing Three Gorges University, Chongqing 404000, China; 3 Spice Crops Research Institute, College of Horticulture and Gardening, Yangtze University, Jingzhou 434023, China; liung906@163.com

**Keywords:** comparative analysis, *A. chinensis* ‘Hongyang’, chloroplast genome, phylogeny

## Abstract

*Actinidia chinensis* ‘Hongyang’, also known as red yangtao (red heart kiwifruit), is a vine fruit tree native to China possessing significant nutritional and economic value. However, information on its genetic diversity and phylogeny is still very limited. The first chloroplast (cp) genome of *A. chinensis* ‘Hongyang’ cultivated in China was sequenced using de novo technology in this study. *A. chinensis* ‘Hongyang’ possesses a cp genome that spans 156,267 base pairs (bp), exhibiting an overall GC content of 37.20%. There were 132 genes that were annotated, with 85 of them being protein-coding genes, 39 transfer RNA (tRNA) genes, and 8 ribosomal RNA (rRNA) genes. A total of 49 microsatellite sequences (SSRs) were detected, mainly single nucleotide repeats, mostly consisting of A or T base repeats. Compared with 14 other species, the cp genomes of *A. chinensis* ‘Hongyang’ were biased towards the use of codons containing A/U, and the non-protein coding regions in the *A. chinensis* ‘Hongyang’ cpDNA showed greater variation than the coding regions. The nucleotide polymorphism analysis (Pi) yielded nine highly variable region hotspots, most in the large single copy (LSC) region. The cp genome boundary analysis revealed a conservative order of gene arrangement in the inverted repeats (IRs) region of the cp genomes of 15 *Actinidia* plants, with small expansions and contractions of the boundaries. Furthermore, phylogenetic tree indicated that *A. chinensis* ‘Hongyang’ was the closest relative to *A. indochinensis*. This research provides a useful basis for future genetic and evolutionary studies of *A. chinensis* ‘Hongyang’, and enriches the biological information of *Actinidia* species.

## 1. Introduction

*Actinidia chinensis* ‘Hongyang’ (*A. chinensis* Planch. var. *chinensis*) is the initial commercially grown cultivar of red-fleshed kiwifruit [1]. Its popularity among consumers stems from its attractive red inner pericarp and exceptional fruit qualities [2]. The kiwifruit fruit has rich nutritional value, and its soluble solid content reaches 19.6%. It also has high levels of vitamin C. Therefore, the kiwifruit is generally referred to as the ‘king of fruit’ [3]. Moreover, the fruits have free radical scavengers, anti-aging, cancer prevention, and other physiological effects favored by the majority of consumers [4]. Although research on kiwifruit chloroplast (cp) genome analysis is rapidly increasing [5,6,7,8,9], little in-depth analysis exists concerning the genetic characteristics and evolution of the commercial variety *A. chinensis* ‘Hongyang’.

Chloroplasts, found in plants, serve as the central hubs for photosynthesis as well as the production of fatty acids, amino acids, and starch. These vital organelles also facilitate the transfer and manifestation of genetic information [10]. Cp genomes are usually between 120 and 170 kb in size and encompass 120–130 genes [11], and cpDNA forms a unique shape resembling a twisted loop, showcasing a distinctive four-part structure. This structure comprises a spacious section known as the large single copy region (LSC), a compact single copy region (SSC), and a pair of inverted repeat (IRs) sequences [12,13,14]. In angiosperms, the cpDNA contains a highly conserved set of protein-coding genes (PCGs), transfer RNA (tRNA) genes, and ribosomal RNA (rRNA) genes [15]. Nevertheless, angiosperm cpDNA has experienced numerous changes due to its ability to adapt to ever-changing surroundings. These alterations encompass not only variations in size and structure but also the contraction and expansion of inverted repeats [16]. Cp genomes are mostly inherited maternally in angiosperms, and molecular phylogenies based on the coding or non-coding areas of cpDNA sequences have proven powerful at the genus and species levels [17]. Due to its relatively stable genome structure and slow rate of nucleotide substitution, cpDNA has gained significant popularity in the fields of plant systematics, phylogeny, and evolution [18].

Advanced sequencing technology has enabled the sequencing and functional characterization of the cpDNAs of numerous species [19]. However, although the cpDNA sequences of some *Actinidia* species have been sequenced, limited knowledge exists regarding the cpDNA information of the cultivated *A. chinensis* ‘Hongyang’ in China. Furthermore, there has been a lack of comprehensive analysis in a single study, particularly concerning the genetic variability and evolutionary correlation with other affiliated species. To delve into the genetic characteristics and evolutionary background of the cultivated *A. chinensis* ‘Hongyang’ and enrich the molecular biological information of the *Actinidia* genus, we performed a comprehensive comparative analysis of *A. chinensis* ‘Hongyang’ cpDNA with other closely associated *Actinidia* species.

The primary aim of this research was to analyze the genomic features of *A. chinensis* ‘Hongyang’ cpDNA, compare it with cpDNA from 14 other *Actinidia* species, and analyze the frequency of codon utilization patterns, in addition to the presence of repeats and small sequence repeats. This would allow us to identify the unique characteristics of the *A. chinensis* ‘Hongyang’ cpDNA. By comparing the complete cpDNA sequences of *A. chinensis* ‘Hongyang’ with those of 28 other *Actinidia* species, we were able to determine their phylogenetic relationships. We have identified potential SSR markers in *Actinidia* plants that could be utilized to investigate genetic variation for DNA barcoding purposes. These findings lay the groundwork for future genomic studies on the genetic variation and evolutionary patterns of *A. chinensis* ‘Hongyang’ and its closely related taxa.

## 2. Materials and Methods

### 2.1. Sample Collection, Extraction of cpDNA, and Sequencing

The newly sprouted foliage of an *A. chinensis* ‘Hongyang’ was gathered from the kiwifruit germplasm resource nursery (29.24 N, 105.87 E) of Chongqing University of Arts and Sciences. We utilized the plant total DNA extraction kit (Beijing Solaibao Technology Co., Ltd., Beijing, China) for the isolation of complete genomic DNA samples. The quality of the DNA products was tested, and the DNA was then fragmented. The short-insert libraries of 350 bp were created using the fragmented DNA, and the libraries that met the quality standards were sequenced on the BGISEQ-500 platform sequencer with PE150 bp, following the instructions provided by the manufacturer.

### 2.2. CpDNA Assembly and Annotation

To trim the raw reads, Fastp [20] was employed, followed by aligning the high-quality reads to the reference cp genomes of *A. chinensis* sourced from GenBank using Bowtie2 v.2.3.4.3 [21]. We utilized NOVOPlasty v4.2.1 [22] to conduct a de novo assembly of the sequence. The annotation of the assembled cp genomes was carried out with the assistance of DOGMA [23], ARAGORN [24], GeSeq [25], and tRNAscan v2.0 [26]. Manual adjustments and confirmations were made using Geneious v9.1.8 [27]. OGDRAW v.1.3.1 [28] was utilized to create the cp genome map. Geneious [27] software was used to extract the LSC, SSC, and IR regions from the cp genome sequences. Statistics on AT and GC content were obtained using BioEdit [29]. The latest cp genome annotation has been uploaded to the GenBank (Number: MW596239).

### 2.3. Analysis of the Cp Genome for Repetitive Sequences 

Employing the REPuter online tool [30], we investigated the occurrence of forward, reverse, palindromic, and complement repeat sequences within the cp genome of *A. chinensis* ‘Hongyang’. The maximum number of repetitive sequences was set to 100, the minimum repetitive element size was 20 bp, the Hamming distance was 3, and the edit distance was selected as the default. The MISA v2.1 software [31] was employed to conduct a search for SSRs. The parameters for mono-, di-, tri-, tetra-, penta-, and hexa-nucleotides were established as 10, 5, 4, 3, 3, and 3, respectively.

### 2.4. Codon Preference Analysis

Codon W v1.4.4 [32] software is employed for codon analysis and the determination of relative synonymous codon usage (RSCU) values for comparative mapping. RSCU represents the relative likelihood of using synonymous codons to encode a specific amino acid; when RSCU > 1, it indicates that the codon is commonly utilized; if RSCU = 1, it implies that the codon lacks any bias in usage; and if RSCU < 1, it signifies that the codon is seldom used.

### 2.5. Sequence Variation Analysis

In order to understand the divergent regions of cp genome sequences in *Actinidia* plants, we utilized the online tool mVISTA [33] to conduct a comprehensive visual alignment analysis on the cp genome data of *Actinidia* plants, encompassing a total of 15 samples, with *A. chinensis* ‘Hongyang’ as the reference sequence. The sliding window size was 100 bp, and the minimum width of the conservative region was 100 bp. The IR region’s expansion and contraction were analyzed using the online tool IRscrope [34]. In order to assess the variability across various regions of the genome, the estimation of nucleotide diversity (Pi) was carried out using DnaSP v5.10 [35]. By employing a window length of 400 bp and a step size of 20 bp, the data were analyzed using the sliding window technique.

### 2.6. Phylogenetic Relationship Analysis

Our study involved analyzing the cp genomes of 29 samples within the Actinidiaceae family using phylogenetic methods. In this analysis, *Clematoclethra scandens*, subsp. *Hemsleyi*, and *Saurauia tristyla* were used as outgroups. The cp genome data of these species were obtained from the GeneBank within the NCBI (Table S1). In order to overcome the difficulties in aligning the entire genome, we utilized Phylosuit v1.1.13 [36] to extract PCG sequences for the phylogenetic analyses. We utilized MAFFT v7.450 [37] for sequence alignment, followed by the removal of regions exhibiting site coverage less than 95% for consistency.

PhyML v2.4.4 [38] and MrBayes v3.2 [39] were utilized to reconstruct maximum likelihood (ML) trees and bayesian (BI) trees, respectively. The determination of nucleotide substitution models was conducted through the utilization of jModelTest v2.1.1 [40]. ML analyses used 1000 bootstrap replicates with the GTR + I + G model.

## 3. Results

### 3.1. Chloroplast Genomic Structure of A. chinensis ’Hongyang’

**Figure 1:**
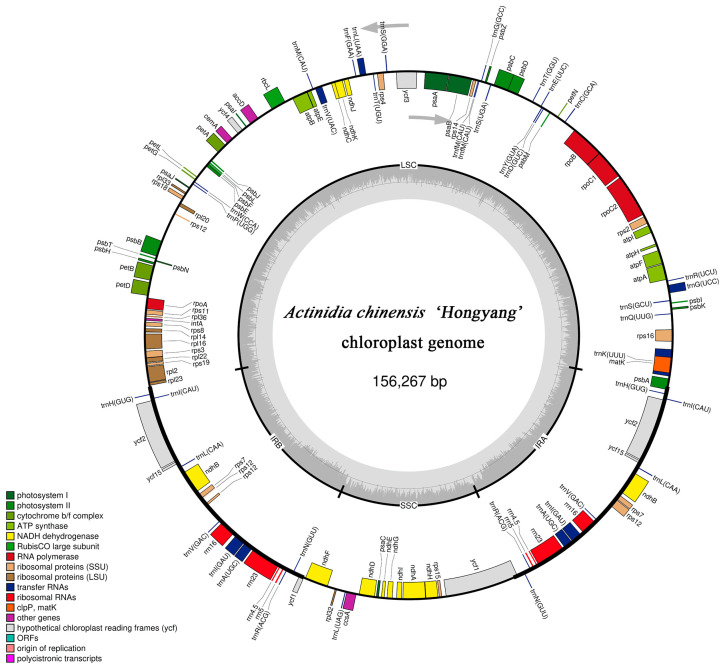
The cp genomes of *A. chinensis* ‘Hongyang’ are depicted in the gene map. The genes located inside the circular structure are transcribed in a clockwise manner, whereas the genes situated outside the circle are transcribed in an anticlockwise manner. Gray arrows represent the direction of gene transcription. Genes with diverse functional groups are visually distinguished by colors. The inner loop of the map, represented by a darker gray color, indicates the GC content. The map displays SSC, LSC, and IR sequences.

The newly sequenced *A. chinensis* ‘Hongyang’ cp genome was 156,267 bp in total length (Figure 1), with the LSC, SSC, and two IRs regions of 87,866 bp, 20,335 bp, and 24,033 bp, respectively. The total GC content of the cp genome of *A. chinensis* ‘Hongyang’ was 37.20%, and the GC contents of the LSC, SSC, and IR regions were 35.30%, 31.15%, and 42.89%, respectively (Table 1). In addition, we compared the cpDNA of *A. chinensis* ‘Hongyang’ and other 14 *Actinidia* species, with genome lengths ranging from 156,124–157,611 bp; the LSC region length range was 86,483–88,666 bp; the SSC region length range was 20,307–22,574 bp; and the IR region length range was 23,377–24,308 bp (Table 2). The comparison revealed that although there were differences in overall genome size, the GC content was similar across species, all around 37.20% (Table 2).

### 3.2. Chloroplast Genome Composition of Actinidia

Based on an in-depth analysis of the cpDNA of *A. chinensis* ‘Hongyang’, 132 genes were identified, including 85 PCGs, 39 tRNA, and 8 rRNA genes (Table 2 and Table 3). These include eight tRNA genes, namely *trnL*(CAA), *trnH*(GUG), *trnI*(CAU), *trnV*(GAC), *trnI*(GAU), *trnA*(UGC), *trnR*(ACG), and *trnN*(GUU); seven PCGs (*psbA*, *ycf2*, *ycf15*, *ndhB*, *rps7*, *rps12*, *ycf1*); and four rRNA genes (*rrn4.5*, *rrn5*, *rrn16*, *rrn23*), which were duplicated in the IR region. The LSC region contains 22 tRNA genes and 61 PCG genes. In contrast, the SSC region of *Actinidia* species contains only 1 tRNA and 10 PCGs. When comparing the total number of PCGs and tRNA genes among 15 *Actinidia* species, there were minimal differences observed. *A. valvata* has a higher number of genes (134); *A. chinensis* has the most PCGs (86); and *A. arguta*, *A. latifolia*, and *A. valvata* have the highest number of tRNA genes. Additionally, *A. chinensis* and *A. rufa* displayed the most abundant PCG content, while *A. arguta*, *A. latifolia*, and *A. valvata* had the highest tRNA content. On the other hand, *A. zhejiangensis* had the lowest tRNA content. The number of rRNA genes in the cpDNA of *Actinidia* plants is relatively conserved and the same as those reported for most angiosperms [41].

Studies have revealed that introns play a crucial role in governing the regulation of gene expression. In plants, numerous introns possess the capacity to amplify the expression of alien genes with precision in terms of timing and spatial positioning, leading to desirable agricultural characteristics [42]. Within the genes encoded by *A. chinensis* ‘Hongyang’, a total of 21 genes have introns. The *ycf3* gene consists of two introns, while *rps12* has two copies. The first exon of *rps12* is present in the LSC region, whereas exons 2 and 3 are located in the IR region. Eight tRNAs (*trnK*(UUU), *trnG*(UCC), *trnL*(UAA), *trnV*(UAC), *trnI*(GAU), *trnA*(UGC), *trnI*(GAU), *trnA*(UGC)), and ten PCGs *(rps16*, *atpF*, *rpoC1*, *petB*, *petD*, *rpl16*, *rpl2*, *ndhB*, *ndhA*, *ndhB)* contain only one intron. The *trnK*(UUU) gene contained the largest intron (2489 bp), and the *trnL*(UAA) gene contained the smallest intron (504 bp) (Table 4).

### 3.3. Codon Usage in the Chloroplast Genome of A. chinensis ’Hongyang’

**Figure 2:**
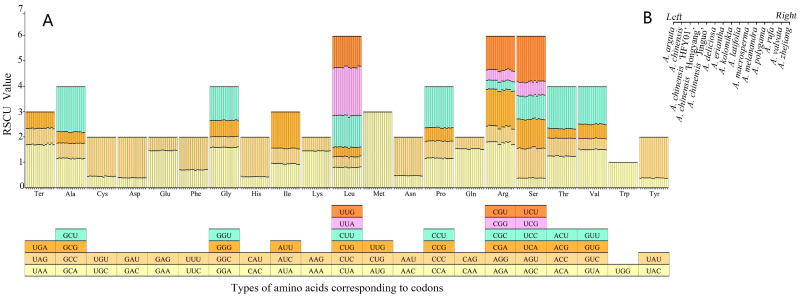
RSCU analysis in cp genes of *Actinidia* plants. (**A**): Preference for codon usage and the proportion of amino acids in the PCGs of the 15 *Actinidia* cp genomes. (**B**): The histogram above each amino acid on the *X*-axis corresponds to the order of the 15 species.

Codons are essential for the correct expression of genetic information. We analyzed and compared the codon usage preferences and RSCU of the cp genome from *A. chinensis* ‘Hongyang’ and its relatives. Based on tRNA and PCG sequences, the frequency of codon usage in the cpDNA of *A*. *chinensis* ‘Hongyang’ was determined and compared with those of 14 closely related plants (Figure 2).The findings indicated that all genes in the cpDNA of *A. chinensis* ‘Hongyang’ were encoded by 25,939 codons; a total of 64 codons were detected, of which UAA, UAG, and UGA were stop codons; and the utilization rate of UAA was high. The highest coding rate among amino acids was for leucine (Leu), which used 2792 codons, accounting for 10.76% of the total, and the lowest coding rate was for cysteine (Cys), which used 278 codons. Of all the codons, AAA was the largest in number, 1114 in total, and the use frequency was 4.29. The number of UGAs and UAGs was the smallest, only 18, and the frequency of usage was 0.07. There were 31 preferred codons (RSCU > 1) in the cp genome of *A. chinensis* ‘Hongyang’. Among the 31 codons, there were twenty-nine codons ending with A/T(U), accounting for 93.55% of the total, two codons ending with G bases (UUG, AUG), and no codons ending with C bases. There existed a pronounced inclination towards the A/U base.

The cp genome codon usage preference analysis of kiwifruit species indicated that the cp genome codon usage preference was basically the same, and they all preferred UAA as the stop codon. Leu, arginine (Arg), and serine (Ser) indicated high codon bias, while tryptophan (Trp) showed no codon bias. Most synonymous codons with RSCU > 1 end with adenine (A) or thymine (U) (except UUG and AUG), showed that there is a high prevalence of codons that conclude with A or U.

### 3.4. Detection of Repeated Sequences and SSRs in A. chinensis ‘Hongyang’ cpDNA

We analyzed the cp genome of *A. chinensis* ‘Hongyang’ and identified a remarkable 99 long repeat sequences. Among them, 39 were found to be palindromic repeats, 4 were reverse repeats, and 56 were forward repeats. Interestingly, no complementary sequences were detected (Table S2). There were 29 repeats of 20–29 bp in length, 23 repeats of 30–39 bp in length, 23 repeats of 40–99 bp in length, and 24 repeats of more than 100 bp in length, and the IR region contains the longest repeated sequence of 287 bp. The majority of the recurring sequences were found within the intergenic spacer region (IGS), while the genes contained only a limited number of recurring sequences (Table S2).

**Figure 3:**
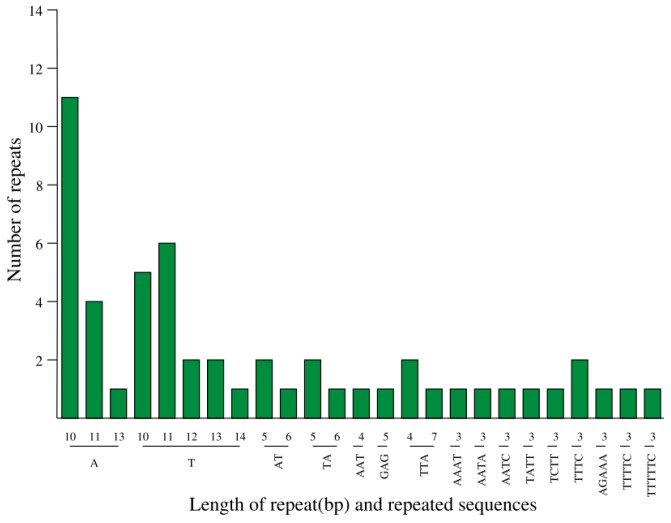
Type and number of SSR nucleotides in the cpDNA of *A. chinensis* ‘Hongyang’.

The SSRs, or microsatellites, with repetitive sequences of 1 to 6 bp of the cp genome of *A. chinensis* ‘Hongyang’ were analyzed. Due to their significant intraspecific polymorphism and maternal inheritance [43,44], SSR markers have gained extensive usage in various fields, such as species identification, population genetics, and evolutionary history research. A total of 49 SSRs were found in *A. chinensis* ‘Hongyang’ cpDNA, including twenty-eight mononucleotides (57.14%), five dinucleotides (10.20%), six trinucleotides (12.24%), two tetranucleotides (4.08%), one pentanucleotide (2.04%), one hexanucleotide (2.04%), and four complex SSRs (8.16%) (Figure 3). Most of the SSRs were found in the LSC region, and they were most densely distributed in the gene spacer (IGS) region (Table S3).

### 3.5. Comparative Analysis of Fifteen Species Chloroplast Genomic Structure

**Figure 4:**
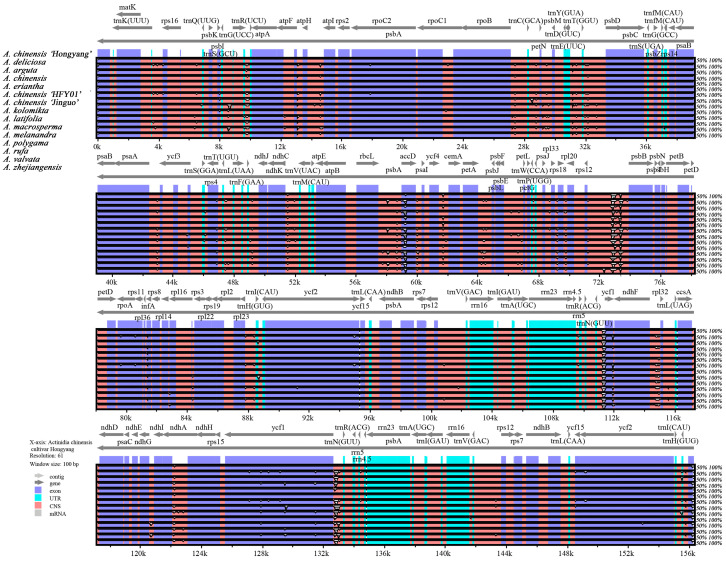
The mVISTA was utilized to analyze and compare the cp genomes of fifteen samples. The gene orientation and the positions of IRs were indicated by gray arrows and bold black lines, respectively. The Y-scale depicted the percentage of identity, ranging from 50% to 100%. To enhance clarity, different genomic regions were color-coded, with protein-coding sequences (exons) shown in blue, rRNA in cyan, and a conserved non-coding sequence (CNS) in pink.

To verify the possibility of genome differentiation, the cp genome of *A. chinensis* ‘Hongyang’ was selected as a reference, and the cp genomes of 15 *Actinidia* plants were visualized and compared using mVISTA (Figure 4). All cp DNA sequences had high sequence consistency and generally showed high interspecific conservation. The coding region displayed a higher level of conservatism compared to the non-coding region, while the inverted repeat region exhibited even greater conservatism than the two single-copy regions. The coding sequences with a high degree of variation were mainly distributed in the *ycf1*, *accD*, *ycf2*, and *rps4* regions. The non-coding regions with a high degree of variation were distributed in the *trnG*(UCC)*-trnR*(UCU), *petN-psdM*, *trnT*(GGU)*-psbD*, *ndhC-trnV*(UAC), *rbcL-accD*, *trnF*(GAA)*-ndhJ*, *rps12-psbB*, *trnC*(GCA)*-petN*, *trnT*(UGU)*-trnL*(UAA), *petA-psbJ*, and *ndhA-ndhH* regions.

**Figure 5:**
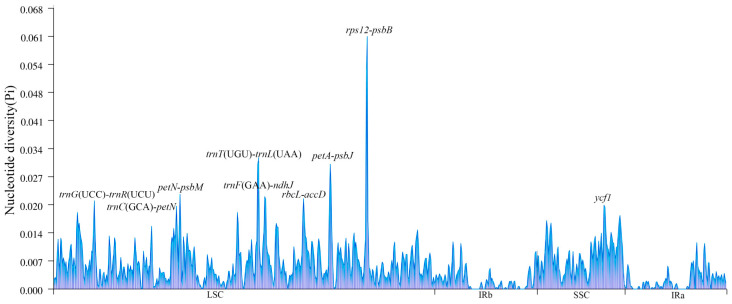
Comparison of nucleotide variability (Pi) values among fifteen different species’cp genomes. The Pi values are depicted on the *Y*-axis, and the genes exhibiting high Pi values are plotted on the *X*-axis.

In addition, researchers determined nucleotide diversity (Pi) values by analyzing 400 bp sections of the *Actinidia* cp genome to pinpoint areas of sequence divergence. The findings revealed a range of Pi values, spanning from 0 to 0.0611, across the 15 genomes studied. We identified nine regions with high variability (Pi > 0.0200): *rps12-psbB*, *petA-psbJ*, *trnT*(UGU)*-trnL*(UAA), *petN-psbM*, *rbcL-accD*, *trnF*(GAA)*-ndhJ*, *trnG*(UCC)*-trnR*(UCU), *trnC*(GCA)*-petN*, and *ycf1* (Figure 5). Out of these, only *ycf1* is situated within the SSC area, while the other eight can be found within the LSC area.

**Figure 6:**
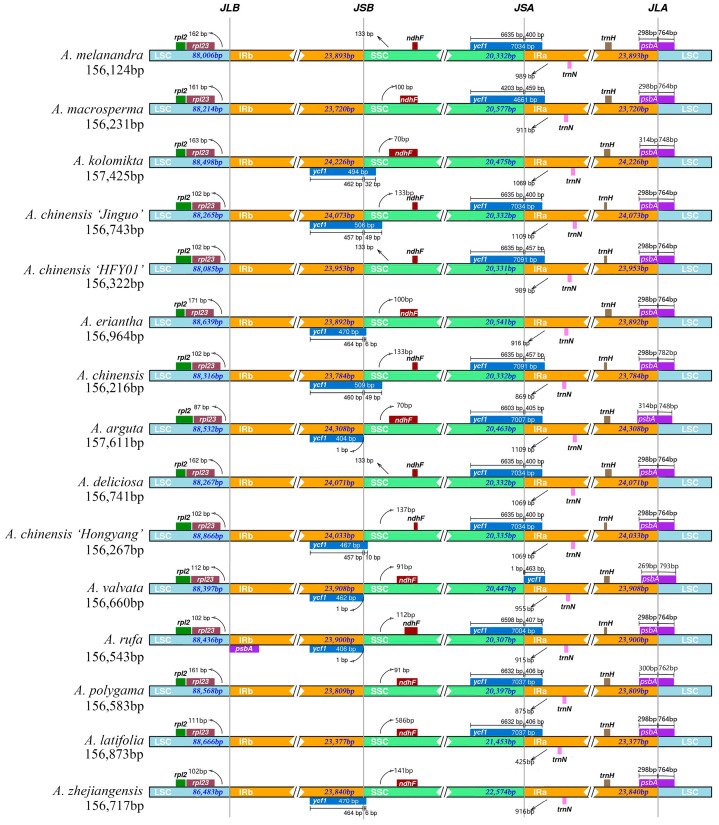
Comparison of the boundaries of LSC, SSC, and IR regions across fifteen cp genomes.

The fluctuation of the IR in plant cp genomes is a widespread occurrence that has significantly influenced species evolution. It is believed to be the primary factor contributing to the varying sizes of plant cpDNA throughout evolution [45] to explore the extent of IR expansion or contraction among *Actinidia* species by comparing the IR/LSC and IR/SSC boundaries of 15 kiwifruit species (Figure 6). It was found that *A. chinensis* ‘Hongyang’ had similar boundaries to *A. chinensis*, *A. chinensis* ‘Jinguo’, and *A. arguta* in the LSC, SSC, and IR regions, but there were still minor differences. Compared to the remaining 11 species, there were large variations in the ranges of each region. All 15 kiwifruit species have *ycf1*, *trnN*, *trnH*, *rpl23*, *ndhF*, and *psbA* genes. The *rpl23* genes were all located in the LSC region and migrated to a lesser extent. The *ndhF* genes were all located in the SSC region, with greater migration of *ndhF* in *A. latifolia* and lesser migration in the remaining species. The *psbA* gene is present across all taxa, extending from the IRa/SSC boundary. It has a varying length of 269–314 bp in the IRa region and 748–793 bp in the LSC region, indicating a contraction of the cpDNA IRa/SSC boundary in *Actinidia* species. The IRb region of *A. rufa* forms the pseudogene *psbA* with incomplete replication. The *ycf1* gene spans the IR/SSC junction of *A. chinensis*, *A. chinensis* ‘Jinguo’, *A. deliciosa*, and *A*. *rufa*, with a small degree of contraction or expansion. The *ycf1* gene of *A. chinensis* ‘HFY01’, *A. macrosperma*, *A. melanandra*, *A. polygama*, *A. deliciosa*, and *A. latifolia* was present only at the IRa/SSC boundary, and the *ycf1* gene of *A. kolomikta*, *A. eriantha*, and *A. zhejiangensis* was present only at the IRb/SSC boundary. Some of these species have incompletely replicated *ycf1* pseudogenes in the IRb region. The *trnN* and *trnH* genes in the cpDNA of *Actinidia* samples were fully replicated at the IR/SSC boundary and are contained in the IR area with a small degree of offset.

### 3.6. Phylogenetic Analysis of 29 Taxa of the Actinidiaceae Based on cpDNA Sequences

**Figure 7:**
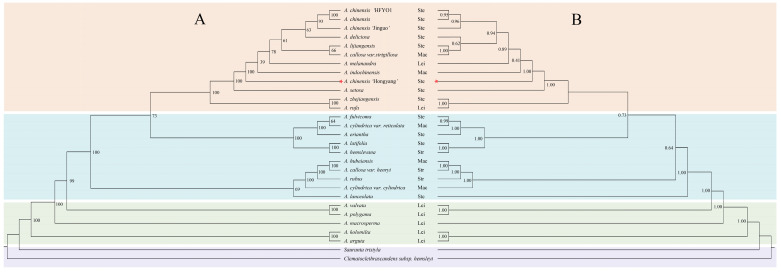
The molecular phylogenetic trees were reconstructed of the genus *Actinidia* using the PCGs data. (**A**): ML tree. (**B**): BI tree. The red pentagrams represent the newly sequenced species in this study. Bootstrap values are indicated by numbers positioned above the branches. Lei: section Leiocarpae Dunn; Ste: section Stellatae; Mac: section Maculatae; Str: section Strigosae.

In order to determine the genetic relationship of *A. chinensis* ‘Hongyang’ in the Actinidiaceae, we used 27 *Actinidia* species and *C*. *scandens* subs. *hemsleyi* and *S*. *tristyla* of the Actinidiaceae as outgroups. The construction of phylogenetic trees employed both maximum likelihood and Bayesian methods. Phylogenetic reliability was tested with 1000 replicates using the bootstrap method. The results indicated that both trees possess an identical complementary structure in terms of topology, except for the terminal branches. The support rate of the branch nodes of the evolutionary tree is basically greater than 60%, and only one node has a value lower than 60%. The high-resolution ML/BI phylogenetic tree indicated that *A. chinensis* ’Hongyang’ was closely related to *A. indochinensis* and *A. setosa* (Figure 7).

## 4. Discussion

The cp genome sequence contains much information that can be used to solve complex evolutionary relationships and has been widely used in plant phylogeny and the evolutionary relationship reconstruction of crop varieties [46]. The genome of *A. chinensis* ‘Hongyang’ exhibits a conventional four-part structure with similar gene composition and high resemblance in PCGs, tRNA, and rRNA, aligning with the cp genomes of other Actinidiaceae species [5]. Structure variations were found in the Actinidiaceae plants’ cp genomes, such as the *clpP* gene, which was deleted, and the *trnfM*-CAU gene in the LSC area, which was a homologous replication [47]. A number of plant lineages have shown that some genes are missing or were transferred to the plant cp genome during the long evolutionary process [48,49,50,51]. However, a comparative study conducted on the cp genome sequences of *A. chinensis* ‘Hongyang’ and 14 other *Actinidia* plants revealed that the *Actinidia* cp genome exhibited a remarkable level of conservation in terms of both genome size and gene content. This finding aligns with previous research conducted at the *Zingiber* [52], *Sinosenecio* [53], and *Camellia* [54] genus levels. Numerous molecular mechanisms have preserved plastid conservatism, such as exclusive inheritance from one parent, the infrequent fusion of plastids, and the presence of efficient repair mechanisms [55,56]. The geographical distribution of the kiwifruit is very wide in East Asia, and the genus is known for its high morphological and ecological variation [57]. Therefore, the maintenance of cp genome sequence conservation may help maintain its normal function and adapt to environmental changes.

The preference for codons is shaped by the forces of natural selection, genetic mutations, and random genetic changes over time. It is a product of the species’ adaptation to their environment through the course of evolution [58]. In this study, the codon preference of *A. chinensis* ‘Hongyang’ was analyzed as a representative of *Actinidia*, and the codon usage preferences and RSCU of *A. chinensis* ‘Hongyang’ and its close relatives’ cpDNA were also compared. The cp genomes of *A. chinensis* ‘Hongyang’ and other *Actinidia* plants are biased toward the use of codons containing A/U. The optimal codons of the plant cp genome can effectively improve the accuracy and efficiency of amino acid translation [59], so the optimal codons of *A. chinensis* ‘Hongyang’ screened in this study can provide a reference for subsequent studies to promote the expression of foreign genes in its chloroplasts.

The polymorphism of SSRs within the cp genome exhibits significant variation among species; these genetic markers have proven to be extremely valuable in this study of population genetics and the understanding of evolutionary processes [60]. The statistical results of SSRs in the *A. chinensis* ’Hongyang’ cp genome showed that the cp genome had a high abundance of SSR types, with the highest percentage of poly T or poly A repeats, while poly C repeats were less common, which was similar to other angiosperms [61]. The discovery of SSR loci in the cp genome of *A. chinensis* ‘Hongyang’ has implications for *Actinidia* plant identification, population-level polymorphism detection, and evolutionary analysis.

The cp genome’s highly diverse regions have the potential to serve not only as markers for species identification and phylogenetic analysis but also as valuable sources of data for investigating population-level species differentiation and population structures [62,63]. In previous studies, fragments such as ITS in the nuclear genome and *matK*, *rbcL*, and *trnH-psbA* in the cp genome have been widely used for interspecific level classification, molecular phylogeny, and DNA barcode applications in higher plants [64]. The vegetative characteristics exhibit a high level of resemblance, which makes distinguishing *Actinidia* plants extremely difficult [65]. Nevertheless, the existing classical DNA barcodes fall short of accurately distinguishing *Actinidia* species and unraveling their evolutionary relationships. Therefore, it is imperative to pinpoint remarkably diverse regions at the genus level, as they hold promising prospects as markers for forthcoming investigations into the identification of different varieties. In light of the findings from mVISTA and the variation in nucleotides, nine significant polymorphisms among fifteen *Actinidia* species were identified, including *rps12-psbB*, *petA-psbJ*, *trnT*(UGU)*-trnL*(UAA), *petN-psbM*, *rbcL-accD*, *trnF*(GAA)*-ndhJ*, *trnG*(UCC)*-trnR*(UCU), *trnC*(GCA)*-petN*, and *ycf1*. The immense variability of these areas could serve as valuable molecular markers for distinguishing *Actinidia* plants and conducting phylogenetic analysis in further studies. Among them, *Actinidia* have been reported to possess *rpl32-trnL-UAG*, *ndhF-rpl32*, and *ycf1* that are suitable for species identification [17,66], but the validity of these fragments for the identification of *Actinidia* plants is still necessary to determine further validation.

The variations in cp genome size are greatly influenced by the expansion and contraction of boundaries within the IR region; these boundary changes are crucial for maintaining structural stability and promoting the evolution of the cp genome [65]. In this research, we found that the cp genomes of 15 *Actinidia* plants, although relatively conserved in structure and size, still showed some variation in the boundary positions of IR and SC between species. The findings are in agreement with a previous report on Actinidiaceae [5]. A shift in the IR/SSC boundary always results in an increase or decrease in the length of the IR area [52]. Many plant species, including *Erodium* [67], *Pilea* [68], and *Pelargonium* [69], exhibited a significant expansion of the IR region, resulting in the incorporation of numerous genes from the SC into this region. In contrast to the cp genomes of 15 *Actinidia* samples, the length of the IR region remained consistent across all species we examined. However, an interesting discovery was made in *A. rufa*, where a pseudogene was observed at the boundary of LSC/IRs. The pseudogene of *ycf1* originated at the junction of IR in *Actinidia* taxa, which was also observed in other angiosperms [50,52,56]. Overall, the different degrees of expansion and contraction of cp genomes at the IR junction of *Actinidia* plants may have been important factors in the formation of polymorphisms in boundaries and genome lengths in *Actinidia* plants during the evolutionary process.

The controversy surrounding the interspecies relationships within the *Actinidia* genus is primarily attributed to the inadequate sampling of taxa and the absence of molecular markers [17]. However, the cp genome offers extensive informative loci for phylogenetic studies. It greatly enhances the accuracy of phylogenetic trees for certain taxonomic groups or those with slow molecular evolution and limited molecular variation. Additionally, it effectively resolves phylogenetic relationships across various taxonomic levels in flowering plants [70]. In contrast to previous studies, the molecular markers of the *Actinidia* species are greatly enhanced in this study [71]. Our study provides a robust phylogenetic hypothesis based on cp genome data that contributes to our understanding of *Actinidia* interspecies relationships (Figure 7). The species-level evolutionary tree of *Actinidia* reveals that the four traditionally recognized sections are not monophyletic with strong support, which is consistent with the results of Tang et al. [17] based on four noncoding cpDNA analyses. However, unlike the outcomes of the analyses of Tang et al. [17], our results strongly supported the notion that *A. zhejiangensis* C.F.Liang is sister to *A. rufa* (Siebold and Zuccarini) Planchon ex. Miquel rather than being grouped with *A. hemsleyana* Dunn; this conflict may be related to the fact that fewer markers were used in their research. As a result, categorizations based on sections fail to accurately represent the authentic evolutionary connections present within this collective. Overall, the robust support for resolving the lineages of *Actinidia* has laid a strong foundation for further classification at the inquisitive level and for exploring the biogeography.

## 5. Conclusions

In this research, the cp genome information of *A. chinensis* ’Hongyang’ was analyzed and annotated. The structural features of the cp genomes of the ‘Hongyang’ kiwifruit variety were found to be remarkably similar to those of 14 other kiwifruit species. Additionally, through the analysis of the cp genomes of 15 different species of *Actinidia*, nine regions were identified as highly variable, making them promising candidates for molecular markers in species identification. The utilization of complete cp genome sequences has effectively elucidated the phylogenetic connections between 29 distinct *Actinidia* species. We found that *A. zhejiangensis* forms a monophyletic group with *A. rufa* rather than being grouped with *A. hemsleyana*, and this conflict may be due to the fact that fewer markers were used in previous research. The findings of our research have confirmed that the sectional categorizations do not reflect the actual evolutionary relationships. Overall, this study has significantly enhanced our knowledge of genome resources while shedding new light on the phylogenetic relationships in *Actinidia* taxonomy.

## Figures and Tables

**Table 1:** Composition of bases in various regions of the cp genome of *A. chinensis* ‘Hongyang’.

Region	A (%)	C (%)	G (%)	T (U) (%)	AT (%)	GC (%)
LSC	31.53	18.16	17.34	32.97	64.50	35.50
SSC	33.83	16.67	14.48	35.02	68.85	31.15
IRs	28.78	20.57	22.31	28.33	57.11	42.89
Total	30.92	18.98	18.23	31.88	62.80	37.20

**Table 2:** Cp genome size and gene number of 15 *Actinidia* species.

Species	GenBank Number	Genome Size (bp)	LSC (bp)	SSC (bp)	IR (bp)	Genes	PCGs	tRNAs	rRNAs	GC Content (%)
*A. chinensis*		156,216	88,316	20,332	23,784	132	86	38	8	37.20
*A. arguta*		157,611	88,532	20,463	24,308	133	84	41	8	37.10
*A. deliciosa*		156,741	88,267	20,332	24,071	130	83	39	8	37.20
*A. eriantha*		156,964	88,639	20,541	23,892	131	84	39	8	37.20
*A. chinensis* ‘HFY01’		156,322	88,085	20,331	23,953	131	84	39	8	37.20
*A. chinensis* ’Jinguo’		156,743	88,265	20,332	24,073	132	85	39	8	37.20
*A. kolomikta*		157,425	88,498	20,475	24,226	131	84	39	8	37.20
*A. latifolia*		156,873	88,666	21,453	23,377	132	83	41	8	37.20
*A. macrosperma*		156,231	88,214	20,577	23,720	132	85	39	8	37.20
*A. melanandra*		156,124	88,006	20,332	23,893	131	84	39	8	37.20
*A. polygama*		156,583	88,568	20,397	23,809	131	83	40	8	37.20
*A. rufa*		156,543	88,436	20,307	23,900	131	84	39	8	37.20
*A. valvata*		156,660	88,397	20,447	23,908	134	85	41	8	37.20
*A. zhejiangensis*		156,717	86,483	22,574	23,840	128	81	39	8	37.20
*A. chinensis* ‘Hongyang’		156,267	87,866	20,335	24,033	132	85	39	8	37.20

**Table 3:** The genes present in *A. chinensis* ‘Hongyang’.

Category	Gene Group	Gene Name	Number
Photosynthesis	Subunits of photosystem I	*psaA*, *psaB*, *psaC*, *psaI*, *psaJ*	5
	Subunits of photosystem II	*psbA*, *psbB*, *psbC*, *psbD*, *psbE*, *psbF*, *psbH*, *psbI*, *psbJ*, *psbK*, *psbL*, *psbM*, *psbN*, *psbT*, *psbZ*	15
	Subunits of NADH dehydrogenase	ndhA *, ndhB *(2), *ndhC*, *ndhD*, *ndhE*, *ndhF*, *ndhG*, *ndhH*, *ndhI*, *ndhJ*, *ndhK*	12
	Subunits of the cytochrome b/f complex	*petA*, petB *, petD *, *petG*, *petL*, *petN*	6
	Subunits of ATP synthase	*atpA*, *atpB*, *atpE*, atpF *, *atpH*, *atpI*	6
	Large subunit of rubisco	*rbcL*	1
Self-replication	Proteins of large ribosomal subunits	*rpl14*, rpl16 *, rpl2 *, *rpl20*, *rpl22*, *rpl23*, *rpl32*, *rpl33*, *rpl36*	9
	Proteins of the small ribosomal subunit	*rps11*, *rps12* **(2), *rps14*, *rps15*, rps16 *, *rps18*, *rps19*, *rps2*, *rps3*, *rps4*, *rps7* (2), *rps8*	14
	Subunits of RNA polymerase	*rpoA*, *rpoB*, rpoC1 *, *rpoC2*	4
	Ribosomal RNAs	*rrn16* (2), *rrn23* (2), *rrn4.5* (2), *rrn5* (2)	8
	Transfer RNAs	*trnA*(UGC) *(2), *trnC*(GCA), *trnD*(GUC), *trnE*(UUC), *trnF*(GAA), *trnG*(GCC), *trnG*(UCC) *, *trnH*(GUG) (2), *trnI*(CAU) (2), *trnI*(GAU) *(2), *trnK*(UUU) *, *trnL*(CAA) (2), *trnL*(UAA) *, *trnL*(UAG), *trnM*(CAU), *trnN*(GUU) (2), *trnP*(UGG), *trnQ*(UUG), *trnR*(ACG) (2), *trnR*(UCU), *trnS*(GCU), *trnS*(GGA), *trnS*(UGA), *trnT*(GGU), *trnT*(UGU), *trnV*(GAC) (2), *trnV*(UAC) *, *trnW*(CCA), *trnY*(GUA), *trnfM*(CAU) (2)	39
Other genes	Maturase	*matK*	1
	Envelope membrane protein	*cemA*	1
	Acetyl-CoA carboxylase	*accD*	1
	c-type cytochrome synthesis gene	*ccsA*	1
	Translation initiation factor	*infA*	1
Genes of unknown function	Conserved hypothetical cp open reading framework	*ycf1* (2), *ycf15* (2), *ycf2* (2), *ycf3* **, *ycf4*	8
Total			132

Notes: *: Gene containing a single intron; **: Gene containing two introns; (2): Number of genes with multiple copies.

**Table 4:** The length of the exon and intron in genes of the *A. chinensis* ‘Hongyang’ cp genome.

Genes	Local	Exon1	Intron1	Exon2	Intron2	Exon3
*rps12*	IRa + LSC	114	536	232		26
*trnK*(UUU)	LSC	37	2489	39		
*rps16*	LSC	38	875	226		
*trnG*(UCC)	LSC	23	689	48		
*atpF*	LSC	145	733	410		
*rpoC1*	LSC	453	748	1605		
*ycf3*	LSC	126	710	228	743	153
*trnL*(UAA)	LSC	37	504	50		
*trnV*(UAC)	LSC	39	595	37		
*rps12-2*	IRb + LSC	114	536	232		26
*petB*	LSC	6	753	645		
*petD*	LSC	8	777	475		
*rpl16*	LSC	9	1055	399		
*rpl2*	LSC	405	664	441		
*ndhB*	IRa	777	679	756		
*trnI*(GAU)	IRa	42	942	35		
*trnA*(UGC)	IRa	38	798	35		
*ndhA*	SSC	553	1085	539		
*trnI*(GAU)-*2*	IRb	42	942	35		
*ndhB-2*	IRb	777	679	756		
*trnA*(UGC)-*2*	IRb	38	798	35		

## Data Availability

The data presented in this study are openly available in GeneBank.

## Supplementary Materials

The following supporting information can be downloaded at: https://www.mdpi.com/article/10.3390/genes14122136/s1, Table S1: Taxonomic and accession information for samples used in this study; Table S2: SSR length and type of *A. chinensis* ‘Hongyang’; Table S3: Repeat sequences in the cp genome of *A. chinensis* ’Hongyang’.

## References

[1] Zhang J.Y., Pan D.L., Jia Z.H., Wang T., Wang G., Guo Z.R. (2018). Chlorophyll, carotenoid and vitamin C metabilism regulation in *Actinidia chinensis* ‘Hongyang’ outer pericarp during fruit development. PLoS ONE.

[2] He J.L., Wu D.T., Zhang Q., Chen H., Li H.Y., Han Q.H., Lai X.Y., Wang H., Wu Y.X., Yuan J.G. (2018). Efficacy and mechanism of cinnamon essential oil on inhibition of colletotrichum acutatum isolated from ‘Hongyang’. Front. Microbiol..

[3] Hunter D.C., Greenwood J., Zhang J.L., Skinner M.A. (2011). Antioxidant and ‘natural protective’ properties of Kiwifruit. Curr. Top. Med. Chem..

[4] Motohashi N., Shirataki Y., Kawase M., Tani S., Sakagami H., Satoh K., Kurihara T., Nakashima H., Mucsi I., Varga A. (2002). Cancer prevention and therapy with kiwifruit in Chinese folklore medicine: A study of kiwifruit extracts. J. Ethnopharmacol..

[5] Yao X.H., Tang P., Li Z.A., Li D.W., Liu Y.F., Huang H.W. (2015). The first complete chloroplast genome sequences in Actinidiaceae: Genome structure and comparative analysis. PLoS ONE.

[6] Wang W.C., Chen S.Y., Zhang X.Z. (2016). Chloroplast Genome Evolution in Actinidiaceae: *clpP* loss, heterogenous divergence and phylogenomic practice. PLoS ONE.

[7] Tang P., Shen R.N., He R.W., Yao X.H. (2019). The complete chloroplast genome sequence of *Actinidia eriantha*. Mitochondrial DNA B.

[8] Lan Y., Cheng L., Huang W., Cao Q., Zhou Z., Luo A., Hu G. (2018). The complete chloroplast genome sequence of *Actinidia kolomikta* from North China. Conserv. Genet. Resour..

[9] Zhang J.L., Liu H. (2019). The complete choloroplast genome sequence of *Actinidia lanceolata*. Mitochondrial DNA B.

[10] Wolfe K.H., Li W.H., Sharp P.M. (1987). Rates of nucleotide substitution vary greatly among plant mitochondrial, chloroplast, and nuclear DNAs. Proc. Natl. Acad. Sci. USA.

[11] Daniell H., Wurdack K.J., Kanagaraj A., Lee S.B., Saski C., Jansen R.K. (2008). The complete nucleotide sequence of the cassava (*Manihot esculenta*) chloroplast genome and the evolution of *atpF* in Malpighiales: RNA editing and multiple losses of a group II intron. Theor. Appl. Genet..

[12] Yue F., Cui L., Depamphilis C.W., Moret B.M.E., Tang J. (2008). Gene rearrangement analysis and ancestral order inference from chloroplast genomes with inverted repeat. BMC Genom..

[13] Aldrich J., Cherney B., Merlin E., Williams C., Mets L. (1985). Recombination within the inverted repeat sequences of the *Chlamydomonas reinhardii* chloroplast genome produces two orientation isomers. Curr. Genet..

[14] Aldrich J., Cherney B.W., Williams C. (1988). Sequence analysis of the junction of the large single copy region and the large inverted repeat in the petunia chloroplast genome. Curr. Genet..

[15] Jian H.Y., Zhang Y.H., Yan H.J., Qiu X.Q., Wang Q.G., Li S.B., Zhang S.D. (2018). The complete chloroplast genome of a key ancestor of modern roses, *Rosa chinensis* var. *spontanea*, and a comparison with congeneric species. Molecules.

[16] Saina J.K., Li Z.Z., Gichira A.W., Liao Y.Y. (2018). The complete chloroplast genome sequence of tree of heaven (*Ailanthus altissima* (Mill.) (*Sapindales:* Simaroubaceae), an important pantropical tree. Int. J. Mol. Sci..

[17] Ping T., Qiang X., Ruinan S., Xiao H.Y. (2019). Phylogenetic relationship in *Actinidia* (Actinidiaceae) based on four noncoding chloroplast DNA sequences. Plant. Syst. Evol..

[18] Ma P.F., Zhang Y.X., Zeng C.X., Guo Z.H., Li D.Z. (2014). Chloroplast phylogenomic analyses resolve deep-level relationships of an intractable bamboo tribe Arundinarieae (Poaceae). Syst. Biol..

[19] Ruban A., Schmutzer T., Scholz U., Houben A. (2017). How next-generation sequencing has aided our understanding of the sequence composition and origin of B chromosomes. Genes.

[20] Chen S., Zhou Y., Chen Y., Gu J. (2018). Fastp: An Ultra-fast All-In-One FASTQ Preprocessor. Bioinformatics.

[21] Langmead B., Salzberg S.L. (2012). Fast gapped-read alignment with Bowtie 2. Nat. Methods.

[22] Dierckxsens N., Mardulyn P., Smits G. (2017). NOVOPlasty: *De novo* assembly of organelle genomes from whole genome data. Nucleic Acids Res..

[23] Wyman S.K., Jansen R.K., Boore J.L. (2004). Automatic annotation of organellar genomes with dogma. Bioinformatics.

[24] Laslett D., Canback B. (2004). ARAGORN, a program to detect tRNA genes and tmRNA genes in nucleotide sequences. Nucleic Acids Res..

[25] Tillich M., Lehwark P., Pellizzer T., Ulbricht-Jones E.S., Fischer A., Bock R., Greiner S. (2017). GeSeq-versatile and accurate annotation of organelle genomes. Nucleic Acids Res..

[26] Chan P.P., Lin B.Y., Mak A.J., Lowe T.M. (2021). tRNAscan-SE 2.0: Improved detection and functional classification of transfer RNA genes. Nucleic Acids Res..

[27] Kearse M., Moir R., Wilson A., Stones-Havas S., Cheung M., Sturrock S., Buxton S., Cooper A., Markowitz S., Duran C. (2012). Geneious basic: An integrated and extendable desktop software platform for the organization and analysis of sequence data. Bioinformatics.

[28] Lohse M., Drechsel O., Bock R. (2007). Organellargenomedraw (ogdraw): A tool for the easy generation of high-quality custom graphical maps of plastid and mitochondrial genomes. Curr. Genet..

[29] Alzohairy A.M. (2011). Bioedit: An important software for molecular biology. GERF Bull. Biosci..

[30] Kurtz S., Choudhuri J.V., Ohlebusch E., Schleiermacher C., Stoye J., Giegerich R. (2001). REPuter: The manifold applications of repeat analysis on a genomic scale. Nucleic Acids Res..

[31] Beier S., Thiel T., Münch T., Scholz U., Mascher M. (2017). MISA-web: A web server for microsatellite prediction. Bioinformatics.

[32] Sharp P.M., Li W.H. (1987). The codon adaptation index—A measure of directional synonymous codon usage bias, and its potential applications. Nucleic Acids Res..

[33] Frazer K.A., Pachter L., Poliakov A., Rubin E.M., Dubchak I. (2004). VISTA: Computational tools for comparative genomics. Nucleic Acids Res..

[34] Amiryousefi A., Hyvönen J., Poczai P. (2018). IRscope: An online program to visualize the junction sites of chloroplast genomes. Bioinformatics.

[35] Librado P., Rozas J. (2009). DnaSP v5: A software for comprehensive analysis of DNA polymorphism data. Bioinformatics.

[36] Zhang D., Gao F.L., Jakovlic I., Zou H., Zhang J., Li W.X., Wang G.T. (2020). PhyloSuite: An integrated and scalable desktop platform for streamlined molecular sequence data management and evolutionary phylogenetics studies. Mol. Ecol. Resour..

[37] Katoh K., Standley D.M. (2013). MAFFT multiple sequence alignment software version 7: Improvements in performance and usability. Mol. Biol. Evol..

[38] Guindon S., Gascuel O. (2003). A simple, fast, and accurate algorithm to estimate large phylogenies by maximum likelihood. Syst. Biol..

[39] Ronquist F., Teslenko M., Van Der Mark P., Ayres D.L., Darling A., Höhna S., Larget B., Liu L., Suchard M.A., Huelsenbeck J.P. (2012). Mrbayes 3.2: Efficient Bayesian phylogenetic inference and model choice across a large model space. Syst. Biol..

[40] Posada D. (2008). jModelTest: Phylogenetic model averaging. Mol. Biol. Evol..

[41] Dong W.P., Xu C., Li D.L., Jin X.B., Li R.L., Lu Q., Suo Z.L. (2016). Comparative analysis of the complete chloroplast genome sequences in psammophytic *haloxylon* species (Amaranthaceae). PeerJ.

[42] Jiao Y., Jia H.M., Li X.W., Chai M.L., Jia H.J., Chen Z., Wang G.Y., Chai C.Y., Weg E.V.D., Gao Z.S. (2012). Development of simple sequence repeat (SSR) markers from a genome survey of Chinese bayberry (*Myrica rubra*). BMC Genom..

[43] Zalapa J.E., Cuevas H., Zhu H., Steffan S., Senalik D., Zeldin E., McCown B., Harbut R., Simon P. (2012). Using next-generation sequencing approaches to isolate simple sequence repeat (SSR) loci in the plant sciences. Am. J. Bot..

[44] Cato S.A., Richardson T.E. (1996). Inter-and intraspecific polymorphism at chloroplast SSR loci and the inheritance of plastids in *Pinus radiata* D. Don. Theor. Appl. Genet..

[45] Wang R.J., Cheng C.L., Chang C.C., Wu C.L., Su T.M., Chaw S.M. (2008). Dynamics and evolution of the inverted repeat-large single copy junctions in the chloroplast genomes of monocots. BMC Evol. Biol..

[46] Somaratne Y., Guan D.L., Wang W.Q., Zhao L., Xu S.Q. (2019). The complete chloroplast genomes of two *Lespedeza* species: Insights into codon usage bias, RNA editing sites, and phylogenetic relationships in Desmodieae (Fabaceae: Papilionoideae). Plants.

[47] Kim S.C., Lee J.W., Baek S.H., Lee M.W., Kang Y.J. (2018). The complete chloroplast genome sequence of *Actinidia rufa* (Actinidiaceae). Mitochondrial DNA B.

[48] Xiong A.S., Peng R.H., Zhuang J., Gao F., Zhu B., Fu X.Y., Xue Y., Jin X.F., Tian Y.S., Zhao W. (2009). Gene duplication, transfer, and evolution in the chloroplast genome. Biotechnol. Adv..

[49] Daniell H., Lin C.S., Yu M., Chang W.J. (2016). Chloroplast genomes: Diversity, evolution, and applications in genetic engineering. Genome Biol..

[50] Li D.M., Li J., Wang D.R., Xu Y.C., Zhu G.F. (2021). Molecular evolution of chloroplast genomes in subfamily Zingiberoideae (Zingiberaceae). BMC Plant. Biol..

[51] Lee C., Ruhlman T.A., Jansen R.K. (2020). Unprecedented intraindividual structural heteroplasmy in *Eleocharis* (Cyperaceae, Poales) plastomes. Genome Biol. Evol..

[52] Jiang D.Z., Cai X.D., Gong M., Xia M.Q., Xing H.T., Dong S.S., Tian S.M., Li J.L., Lin J.Y., Liu Y.Q. (2023). Complete chloroplast genomes provide insights into evolution and phylogeny of *Zingiber* (Zingiberaceae). BMC Genom..

[53] Peng J.Y., Zhang X.S., Zhang D.G., Wang Y., Deng T., Huang X.H., Kuang T.H., Zhou Q. (2022). Newly reported chloroplast genome of *Sinosenecio albonervius* y. Liu & Q.E. Yang and comparative analyses with other *Sinosenecio* species. BMC Genom..

[54] Li L., Hu Y.F., He M., Zhang B., Wu W., Cai P., Huo D., Hong Y.C. (2021). Comparative chloroplast genomes: Insights into the evolution of the chloroplast genome of *Camellia sinensis* and the phylogeny of *Camellia*. BMC Genom..

[55] Wicke S., Schneeweiss G.M., Depamphilis C.W., Müller K.F., Quandt D. (2011). The evolution of the plastid chromosome in land plants: Gene content, gene order, gene function. Plant. Mol. Biol..

[56] Abdullah, Mehmood F., Rahim A., Heidari P., Ahmed I., Poczai P. (2021). Comparative plastome analysis of *Blumea*, with implications for genome evolution and phylogeny of Asteroideae. Ecol. Evol..

[57] Liu Y.F., Li D.W., Zhang Q., Song C., Zhong C.H., Zhang X.D., Wang Y., Yao X.H., Wang Z.P., Zeng S.H. (2017). Rapid radiations of both kiwifruit hybrid lineages and their parents shed light on a two-layer mode of species diversification. New. Phytologist..

[58] Xiong B., Wang T., Huang S.J., Liao L., Wang X., Deng H.H., Zhang M.F., He J.X., Sun G.C., He S.Y. (2023). Analysis of codon usage bias in xyloglucan endotransglycosylase (XET) genes. Int. J. Mol. Sci..

[59] Parvathy S.T., Udayasuriyan V., Bhadana V. (2022). Codon usage bias. Mol. Biol. Rep..

[60] Nie X.J., Lv S.Z., Zhang Y.X., Du X.H., Wang L., Biradar S.S., Tan X.F., Wan F.H., Weining S. (2012). Complete chloroplast genome sequence of a major invasive species, crofton weed (*Ageratina adenophora*). PLoS ONE.

[61] Kuang D.Y., Wu H., Wang Y.L., Gao L.M., Zhang S.Z., Lu L. (2011). Complete chloroplast genome sequence of *Magnolia kwangsiensis* (Magnoliaceae): Implication for DNA barcoding and population genetics. Genome.

[62] Dang Y.Y., Yang Y., Li Q., Lu J.J., Li X.W., Wang Y.T. (2014). Complete chloroplast genome sequence of poisonous and medicinal plant *Datura stramonium*: Organizations and implications for genetic engineering. PLoS ONE.

[63] Chen Q., Wu X.B., Zhang D.Q. (2019). Phylogenetic analysis of *Fritillaria cirrhosa* D. Don and its closely related species based on complete chloroplast genomes. PeerJ.

[64] Chase M.W., Cowan R.S., Hollingsworth P.M., Berg C., Wilkinson M.J. (2007). A proposal for a standardised protocol to barcode all land plants. Taxon.

[65] He L., Qian J., Li X.W., Sun Z.Y., Xu X.L., Chen S.L. (2017). Complete chloroplast genome of medicinal plant *Lonicera japonica*: Genome rearrangement, intron gain and loss, and implications for phylogenetic studies. Molecules.

[66] Xie Q.L., Zhang H.B., Yan F., Yan C.X., Wei S.G., Lai J.H., Wang Y.P., Zhang B. (2019). Morphology and molecular identification of twelve commercial varieties of Kiwifruit. Molecules.

[67] Blazier J.C., Jansen R.K., Mower J.P., Govindu M., Zhang J., Weng M.L., LRuhlman T.A. (2016). Variable presence of the inverted repeat and plastome stability in *Erodium*. Ann. Bot..

[68] Li J.L., Tang J.M., Zeng S.Y., Han F., Yuan J., Yu J. (2021). Comparative plastid genomics of four *Pilea* (Urticaceae) species: Insight into interspecifc plastid genome diversity in *Pilea*. BMC Plant Biol..

[69] Weng M.L., Ruhlman T.A., Jansen R.K. (2017). Expansion of inverted repeat does not decrease substitution rates in *Pelargonium* plastid genomes. New. Phytol..

[70] Zhang X., Zhou T., Kanwal N., Zhao Y.M., Bai G.Q., Zhao G.F. (2017). Completion of eight *Gynostemma* bl. (Cucurbitaceae) chloroplast genomes: Characterization, comparative analysis, and phylogenetic relationships. Front. Plant. Sci..

[71] Chat J., Jáuregui B., Petit R.J., Nadot S. (2004). Reticulate evolution in kiwifruit (*Actinidia*, Actinidiaceae) identified by comparing their maternal and paternal phylogenies. Am. J. Bot..

